# Auswirkungen von hohen Außentemperaturen und Hitzewellen auf Lungenerkrankungen: Rolle von Pneumolog:innen beim gesundheitlichen Hitzeschutz

**DOI:** 10.1007/s10405-023-00500-5

**Published:** 2023-03-24

**Authors:** F. Matthies-Wiesler, N. Nidens, S. Karrasch, A. Schneider

**Affiliations:** 1 Institut für Epidemiologie, Helmholtz Zentrum München – Deutsches Forschungszentrum für Gesundheit und Umwelt (GmbH), Ingolstädter Landstr. 1, 85764 Neuherberg, Deutschland; 2 KLUG – Deutsche Allianz Klimawandel und Gesundheit e. V., Cuvrystr. 1, 10997 Berlin, Deutschland; 3 Institut und Poliklinik für Arbeits‑, Sozial- und Umweltmedizin, Klinikum der Universität München, LMU München, München, Deutschland

**Keywords:** Klimawandel, Atemwegserkrankungen, Herz-Kreislauf-Erkrankungen, Gesundheitsbezogene Hitzeschutzmaßnahmen, Gesundheitsrisiko, Climate change, Respiratory diseases, Cardiovascular diseases, Health-related heat protection measures, Health risk

## Abstract

**Hintergrund:**

Der fortschreitende Klimawandel führt zu häufigeren und intensiveren Hitzewellen. Im Vergleich zu 1951 erlebt Deutschland bereits heute im Durchschnitt nicht nur etwa 3 heiße Tage im Sommer, sondern 8,8 heiße Tage. Für die Sommer 2018, 2019, 2020 und 2022 wurden insgesamt etwa 23.800 hitzebedingte Todesfälle durch Modellierung der Übersterblichkeit berechnet. Für vulnerable Bevölkerungsgruppen stellen Hitzewellen ein erhöhtes Gesundheitsrisiko dar. Zu ihnen gehören ältere Menschen und Menschen mit Vorerkrankungen sowie Säuglinge, Schwangere und Personen, die im Freien körperlich schwer arbeiten oder Sport treiben. Hitze kann zu Hitzeerschöpfung und lebensbedrohlichem Hitzschlag führen und Herzinfarkte und Schlaganfälle auslösen.

**Problemstellung:**

Menschen mit Atemwegserkrankungen sind besonders betroffen, wenn die hohen Temperaturen zusätzlich mit erhöhter Luftverschmutzung einhergehen. Hitzebedingte Lungenprobleme wie eine erhöhte pulmonale Belastung etwa durch hitzebedingte Hyperventilation und erhöhte Luftverschmutzung sowie mit kardialer Beeinträchtigung und Pneumonien assoziierte Effekte erhöhen das Risiko für Mortalität und Morbidität während Hitzewellen für betroffene Patient:innen.

**Schlussfolgerungen:**

Pneumolog:innen können durch hitzespezifische Beratung und Behandlung einen maßgeblichen Beitrag zu gesundheitsbezogenem Hitzeschutz leisten. Sie sind daher aufgefordert, sich in ihrem Fachbereich entsprechend zu informieren und Hitzeschutzmaßnahmen zum Schutz ihrer Patient:innen und Mitarbeiter:innen in Praxen und Krankenhausabteilungen umzusetzen.

## Abstract (en)

**Background:**

The progressing climate change leads to more frequent and more intense heat waves. Compared to 1951, Germany now experiences on average 8.8 hot days over the summer instead of 3 hot days. For the summers of 2018, 2019, 2020 and 2022 a cumulative number of 23,800 heat-related deaths were calculated based on excess mortality modelling. For vulnerable population groups, heat waves pose an increased risk to health. These groups include the elderly, people with chronic diseases, infants, pregnant women, and people who perform physically hard work or carry out sports outdoors. High temperatures can lead to heat exhaustion and life-threatening heatstroke as well as triggering myocardial infarction and stroke.

**Objective:**

Individuals with chronic pulmonary diseases are particularly affected when high temperatures coincide with high levels of air pollution. Heat-related lung problems, such as increased pulmonary load through heat-related hyperventilation together with increased air pollution as well as effects associated with cardiac impairment and pneumonia increase the risk of morbidity and mortality for affected patients during heat waves.

**Conclusion:**

Pulmonologists can contribute to health-related heat protection for their patients through heat-specific advice and treatment. Pulmonologists are therefore required to be well-informed regarding measures in their field and to implement heat protection measures for the protection of their patients and employees in their practices and hospital departments.

Durch den Klimawandel erhöhen sich Häufigkeit und Intensität von Hitzewellen in Deutschland. Gerade für chronisch Vorerkrankte können hohe Temperaturen gesundheitliche Auswirkungen haben. Hitzebedingte Lungenprobleme wie eine erhöhte pulmonale Belastung durch hitzebedingte Hyperventilation und erhöhte Luftverschmutzung führen zu einem höheren Risiko für Mortalität und Morbidität während Hitzewellen für betroffene Patient:innen. Pneumolog:innen können durch hitzespezifische Beratung und Behandlung einen maßgeblichen Beitrag zu gesundheitsbezogenem Hitzeschutz leisten.

## Klimawandelfolgen: Es wird heiß in Deutschland

Der Klimawandel birgt über direkte und indirekte Effekte die größte Herausforderung für die Gesundheit im 21. Jahrhundert. Steigende Temperaturen können die Ausbreitung von Infektionskrankheiten begünstigen, die durch Vektoren (z. B. Mücken oder Zecken) übertragen werden. Das kann Infektionskrankheiten betreffen, die hierzulande bereits vorkommen (z. B. Frühsommermeningoenzephalitis [FSME] oder Borreliose) [2], aber auch solche, die vor Ort noch nicht übertragen werden (z. B. Denguefieber, Zika oder Chikungunya) [34]. Auch die Biologie allergener Pollen verändert sich mit steigenden Temperaturen, sodass sich die saisonale Dauer des Pollenflugs verlängert und die Pollenmenge ansteigt [40]. Mit dem Klimawandel verbundene Extremwetterereignisse wie Hitzewellen und Überschwemmungen zeigen direkte Auswirkungen auf die Gesundheit. Vor allem die zunehmende Hitze stellt für Menschen in Deutschland das größte Risiko dar [11, 25]. Die Anzahl an heißen Tagen mit Temperaturen über 30 °C steigt, schon heute gibt es ca. 8,8 heiße Tage anstelle von 3 heißen Tagen, wie es in den 1950er-Jahren hierzulande war [11]. Bis zum Ende des Jahrhunderts werden bei gleichbleibenden Treibhausgasemissionen in Norddeutschland ca. 5 bis 10 zusätzliche heiße Tage erwartet, im Süden ca. 10 bis 15 [32]. Mit voranschreitendem Klimawandel sind häufigere, längere und intensivere Hitzewellen zu erwarten. Auch gegen extreme Hitzeereignisse bisher nicht bekannten Ausmaßes muss sich Deutschland noch in dieser Dekade wappnen.

Unter einem „Weiter-so“-Szenario kann es bis Ende des Jahrhunderts bis 54 heiße Tage im Jahr geben [32].

## Auswirkungen von hohen Temperaturen und Hitzewellen auf die Gesundheit

**Figure 1:**
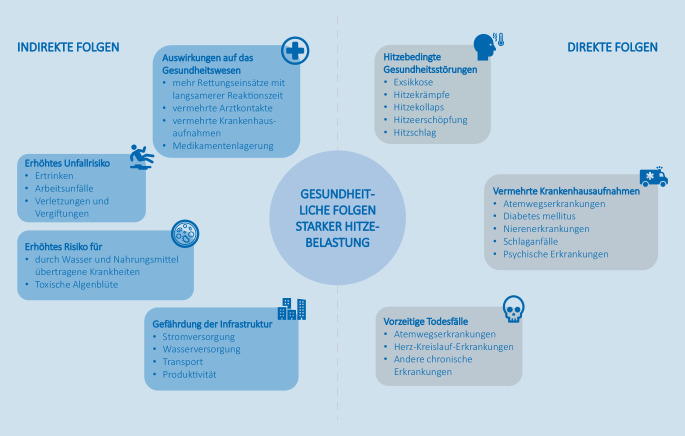

Hohe Temperaturen haben bereits ernsthafte Folgen für die Gesundheit von Millionen von Menschen (Abb. 1). Zu den vulnerablen Bevölkerungsgruppen gehören v. a. Ältere und Vorerkrankte, beispielsweise mit kardiovaskulären, respiratorischen oder psychischen Erkrankungen und Nierenfunktionsstörungen. Doch auch die Gesundheit von Kleinkindern, Schwangeren und im Freien Arbeitenden oder Sportler:innen ist gefährdet (Tab. 1). Hitzeassoziierte Erkrankungen und Todesfälle betreffen aufgrund der Anpassungsfähigkeit und -möglichkeit häufig alleinstehende, sozial isolierte Menschen, Obdachlose und Menschen in ungünstigen Wohnsituationen [36].

**Table 1:** 

Kategorie	Risikofaktor
Alter	Ältere Menschen (> 65 Jahre)
	Säuglinge und Kleinkinder
Vorerkrankungen	Kardiovaskuläre Erkrankungen (z. B. arterielle Hypertonie, koronare Herzkrankheit, Herzinsuffizienz)
	Zerebrovaskuläre Erkrankungen
	Respiratorische Erkrankungen (z. B. COPD, Asthma bronchiale)
	Stoffwechselerkrankungen (z. B. Diabetes mellitus)
	Neurologische Erkrankungen (z. B. Morbus Parkinson)
	Psychische Erkrankungen
	Nierenerkrankungen (z. B. Niereninsuffizienz)
	Übergewicht
	*Einnahme von bestimmten Medikamenten zur Behandlung der genannten Erkrankungen, z.* *B.:* Antiadrenergika Anticholinerge Arzneimittel (z. B. Antipsychotika, trizyklische Antidepressiva, Antiparkinsonmittel, Antihistaminika) Antihypertensiva Anxiolytika und Muskelrelaxanzien Diuretika Antianginosa Antiepileptika Schmerzmittel (z. B. NSAR, Opioide) Insuline
Funktionelle Einschränkungen	Bettlägerigkeit
	Unterbringung in Pflegeeinrichtung
Sozioökonomische Faktoren	Alleinstehende Menschen, insbesondere im hohen Alter
	Soziale Isolation
	Obdachlosigkeit
	Ungünstige Wohnsituation

*COPD* chronisch obstruktive Lungenerkrankung, *NSAR* nichtsteroidale Antirheumatika

**Figure 2:**
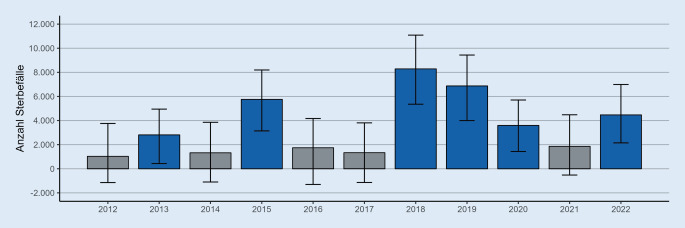

In Deutschland versterben jährlich mehrere hundert bis mehrere tausend Menschen im Zusammenhang mit Hitze. Allein während der Hitzewelle im Jahr 2003 kam es laut Modellierung der Mortalitätsrate im Zusammenhang mit der Wochenmitteltemperatur zu 9500 Todesfällen, aber auch für die Sommer in jüngeren Jahren wurden immer wieder über 5000 Todesfälle berechnet [38]. Eine Untersuchung von 2022 zeigt, dass von 2018 bis 2020 eine signifikante hitzebedingte Übersterblichkeit mit insgesamt 19.300 Todesfällen in 3 aufeinanderfolgenden Jahren in Deutschland aufgetreten ist ([38]; Abb. 2). Auch für den Sommer 2022, den sonnigsten und einen der 4 wärmsten seit Aufzeichnungsbeginn im Jahr 1881 [19, 20], wurden deutschlandweit ca. 4500 hitzebedingte Todesfälle geschätzt [37], wobei bei dieser Betrachtung die Auswirkung auf die Morbidität nicht einbezogen wird.

Als Ursache für hitzebedingte Todesfälle kommt Hitzschlag in Betracht, aber auch die Exazerbation von kardiovaskulären oder respiratorischen Vorerkrankungen kann ein Faktor sein. Neben hitzebedingten Todesfällen kommt es auch zu hitzebedingten Erkrankungen, z. B. zu Hitzeerschöpfung, und Komplikationen aufgrund von Vorerkrankungen und entsprechender Medikation [36]. Das Gesundheitssystem wird während Hitzeperioden durch die vermehrten Krankenhauseinweisungen und Rettungsdiensteinsätze verstärkt in Anspruch genommen – wobei auch das medizinische Personal selbst z. T. unter anstrengenden Bedingungen bei hohen Temperaturen und oft in persönlicher Schutzausrüstung arbeitet.

## Hitzebedingte respiratorische Beeinträchtigungen

**Figure 3:**
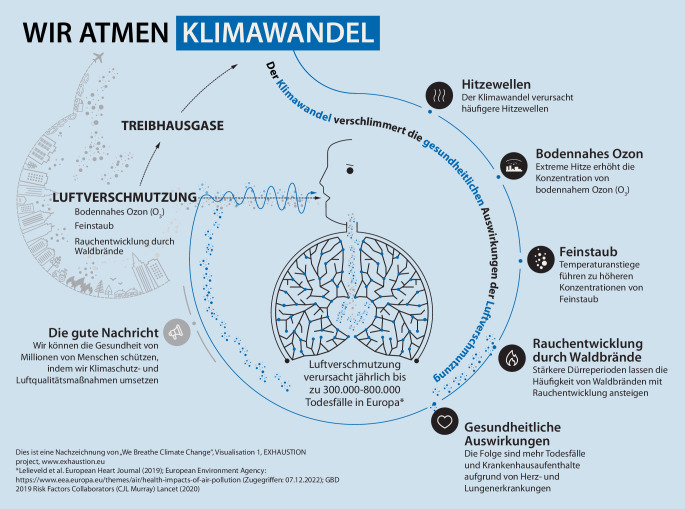

Bei hitzebedingter Morbidität und Mortalität von Atemwegserkrankungen sind die zugrunde liegenden Mechanismen bisher noch nicht gut verstanden, und sie treten oft in Kombination mit kardiovaskulären Effekten auf. Primär scheinen Menschen mit bereits bestehenden Atemwegserkrankungen betroffen zu sein, und die chronisch obstruktive Lungenerkrankung (COPD) ist in der älteren Bevölkerung einer der häufigsten Gründe für Krankenhauseinweisungen aufgrund von Atemwegserkrankungen in Verbindung mit Hitzeexposition (Abb. 3; [31, 35]). Bei Patient:innen mit COPD kann durch Hyperventilieren während extremer Hitzeereignisse [33] das Risiko einer dynamischen Hyperinflation steigen, und daraufhin können Dyspnoe und mechanische bzw. kardiovaskuläre Effekte auftreten. Beobachtungen zeigen, dass es in den Sommermonaten an heißen Tagen häufiger zu teils lebensbedrohlichen Exazerbationen von Patient:innen mit COPD kommt, obwohl diese typischerweise ansonsten im Winter häufiger sind [12].

Lungenprobleme zählen zu den häufigsten Ursachen für Mortalität und Morbidität während Hitzewellen

In einer neuen Studie wurden die Zusammenhänge zwischen Lufttemperatur und ursachenspezifischer Mortalität bzw. Krankenhauseinweisungen aufgrund von Atemwegserkrankungen in der warmen Jahreszeit (Mai bis September) in Deutschland auf kleinräumiger Ebene untersucht. Von 2000 bis 2016 wurden tägliche Daten im Besonderen zu COPD, Asthma, Infektionen der unteren Atemwege und Pneumonien erhoben. Dabei wurden erhöhte Risiken für alle untersuchten ursachenspezifischen respiratorischen Mortalitäten und Krankenhauseinweisungen im Zusammenhang mit Hitze beobachtet, u. a. starke Effekte in Bezug auf Pneumonien [6]. Die Mechanismen sind noch nicht vollends verstanden.

Grundsätzlich könnte ein weiterer möglicher Faktor für den Zusammenhang zwischen hohen Lufttemperaturen und Atemwegserkrankungen sein, dass bei Hitze eine Wärmeabgabe nicht nur über die Haut, sondern auch über die Lunge erfolgt, wobei sich die Atemfrequenz leicht erhöht. Dieser Wärmetransport kann bei Atemwegserkrankungen allerdings beeinträchtigt und damit erschwert sein [39].

## Hitze und Luftverschmutzung wirken gemeinsam

Hohe Lufttemperaturen in Verbindung mit intensiver Sonneneinstrahlung begünstigen zum einen die Bildung des Luftschadstoffs Ozon (O_3_), zum anderen kann sich die Feinstaubbelastung durch Entstehung von sog. sekundären Aerosolen erhöhen. Natürliche Prozesse wie Vegetationsbrände und die Windverfrachtung staubtrockenen Bodens während lang anhaltender sommerlicher Trockenheit können eine erhebliche Zusatzbelastung der Gesamt(fein)staubemission sein. Zudem halten sich die Menschen in der warmen Jahreszeit mehr im Freien auf bzw. haben die Fenster geöffnet und sind deshalb auch gegenüber Hitze und Luftschadstoffen verstärkt exponiert.

Patient:innen mit kardiometabolischen und respiratorischen Erkrankungen sind durch Umweltstressoren besonders gefährdet. Steigende Temperaturen, extreme Hitzeereignisse, aber auch Luftverschmutzung (z. B. Feinstaub, Ozon, Stickstoffdioxid) können bei diesen Patientengruppen zu akuten, möglicherweise auch lebensbedrohlichen Ereignissen führen [4, 7, 10, 13, 17]. Während einer Hitzewelle oder bei schlechter Luftqualität wird z. B. eine Erhöhung von Blutdruck und Herzfrequenz beobachtet, einhergehend mit einem erhöhten Risiko für akute Ereignisse wie etwa Herzinfarkt oder Schlaganfall [7, 9, 13]. Umweltfaktoren können aber auch über längere Zeiträume hinweg durch Stimulation entzündlicher Prozesse bzw. durch oxidativen Stress Erkrankungen begünstigen [29]. Potenzielle Pathomechanismen legen nahe, dass es bei vielen regulatorischen Abläufen im Körper Parallelen zwischen den Einflüssen der beiden Umweltfaktoren gibt. Somit ist vorstellbar, dass es hier zu Interaktionen und Synergien von Lufttemperatur und Luftschadstoffen kommt [7].

Bisher wurden hohe Lufttemperaturen bzw. Hitzeereignisse und Luftschadstoffe meist getrennt voneinander betrachtet. So wurden zwar in vielen Studien die gesundheitlichen Auswirkungen verschiedener Luftschadstoffe untersucht, indem die Lufttemperatur als potenzieller Störfaktor berücksichtigt wurde, und auch umgekehrt wurden verschiedenste Luftschadstoffe bereits als potenzielle Störgrößen im Zusammenhang zwischen Lufttemperatur und Gesundheit miteinbezogen. Hingegen wurden die Wechselwirkungen zwischen Hitze und Luftschadstoffen sowie ihre kombinierten Auswirkungen auf den Menschen noch nicht ausreichend erforscht [1, 7].

## Hitze verstärkt die Wirkung von Luftschadstoffen und umgekehrt

Die meisten solcher Studien untersuchten die Veränderung der Auswirkungen von Luftschadstoffen durch die Temperatur, wobei die Mehrzahl dieser Studien zeigte, dass hohe Temperaturen die Effekte von Ozon oder Feinstaub auf die (ursachenspezifische) Mortalität verstärken [30]. Im Gegensatz dazu sind Untersuchungen der Frage, ob die Luftverschmutzung die Auswirkungen der Temperatur modifiziert, immer noch begrenzt [7, 24]. Hier kann das laufende EU-Projekt EXHAUSTION (https://www.exhaustion.eu/) einige Forschungslücken schließen. In den Analyseergebnissen zeigte sich ein erhöhtes Risiko für kardiovaskuläre und respiratorische Todesfälle und Krankenhausaufenthalte im Zusammenhang mit Hitzeexposition in der warmen Jahreszeit, und es stellte sich die Frage, ob diese Hitzewirkung durch Luftschadstoffe verstärkt wird (Abb. 3; [23]). In dem Projekt wurden daher Wirkungen von Feinstaub, also Partikel mit einem Durchmesser ≤ 2,5 μm (PM_2.5_), und Wirkungen des Reizgases Ozon (O_3_) untersucht. Es wurde eine deutliche Wechselwirkung zwischen hohen Lufttemperaturen und PM_2.5_ beobachtet mit den höchsten hitzebedingten Auswirkungen auf die Mortalität und erhöhten Hospitalisierungsraten an Tagen mit gleichzeitig hohen PM_2.5_-Werten. Zudem wurden Hinweise auf Wechselwirkungen zwischen hohen Temperaturen und O_3_ für kardiovaskuläre und respiratorische Todesfälle, aber nicht für Krankenhauseinweisungen gefunden mit dem höchsten Anstieg der Mortalität an Tagen mit hohen O_3_-Belastungen.

Während die meisten bisherigen Arbeiten die gesundheitliche Wirkung des Zusammenspiels von kurzzeitiger Exposition gegenüber Lufttemperatur und Luftschadstoffen untersuchten, gibt es bislang nur sehr wenige Studien zum Zusammenspiel von chronischer Belastung durch Luftschadstoffe und Lufttemperatur (z. B. [26]). Angesichts der erwarteten klimatischen Veränderungen ist es von Bedeutung, auch die längerfristigen Auswirkungen, wie etwa jährliche Durchschnittstemperaturen, und ihr Zusammenspiel mit chronischer Luftschadstoffbelastung zu verstehen.

## Gesundheitsbezogener Hitzeschutz – das Beispiel „Aktionsbündnis Hitzeschutz Berlin“

Obwohl 2017 das Bundesumweltministerium Handlungsempfehlungen zur Erstellung von Hitzeaktionsplänen zum Schutz der menschlichen Gesundheit veröffentlichte [8] und die gemeinsame Gesundheitsministerkonferenz (GMK) 2020 die Erstellung von Hitzeaktionsplänen innerhalb eines 5‑Jahres-Zeitraums fordert [16], ist Deutschland laut dem Lancet Countdown Policy Brief für Deutschland 2021 bisher nicht ausreichend auf Hitzewellen vorbereitet [21]. Obwohl es dringend nötig ist, Hitzeschutzaktionspläne auf Landes- und kommunaler Ebene umfassend umzusetzen [18], wurden Hitzeaktionspläne bisher nur in wenigen Kommunen eingeführt, und auch im medizinischen Sektor besteht noch Entwicklungsbedarf. So haben nur wenige Krankenhäuser eigene Hitzeschutzpläne, und auch in der Aus‑, Fort- und Weiterbildung erscheinen hitzebedingte Gesundheitsrisiken unterrepräsentiert. Aus den Empfehlungen des Lancet Countdown Policy Briefs für Deutschland 2021 lässt sich daher ableiten, dass Gesundheitseinrichtungen an das Frühwarnsystem des Deutschen Wetterdienstes (DWD) angeschlossen werden und institutionelle Maßnahmen zur strukturellen und organisatorischen Vorbereitung auf Hitzewellen erstellt werden sollten.

Deutschland ist bisher nicht ausreichend auf Hitzewellen vorbereitet

Ärzt:innen tragen die Verantwortung, Gesundheit zu schützen und zu erhalten. Diese Verantwortung schließt auch Hitze als klimawandelbedingtes Gesundheitsrisiko für Menschen in Deutschland ein. Ärzt:innen können bereits mit kurzfristigen, schnell umsetzbaren Maßnahmen zur Risikominimierung für vulnerable Bevölkerungsgruppen beitragen und sind daher essenziell für einen funktionierenden Hitzeschutz.

**Figure 4:**
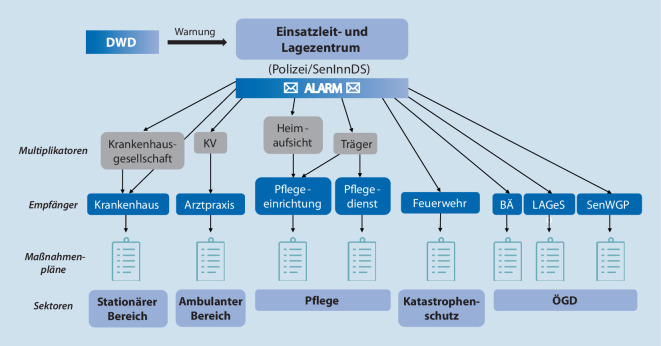

Ein Beispiel dafür, wie sich das Gesundheitswesen aufstellen kann, zeigt das „Aktionsbündnis Hitzeschutz Berlin“. In dem im März 2022 gegründeten Netzwerk arbeiten Ärzt:innenschaft, Pflege, Katastrophenschutz, Rettungsdienste sowie der Öffentliche Gesundheitsdienst zusammen, um zum Schutz vulnerabler Bevölkerungsgruppen in akuten Hitzesituationen beizutragen. So wurde im Sommer 2022 eine Warnkette für anstehende Hitzeereignisse implementiert (Abb. 4), Musterhitzeschutzpläne für Gesundheitseinrichtungen mit beispielhaften Hitzeschutzmaßnahmen wurden erstellt und Informations- und Schulungsmaterialien entwickelt (s. Infobox). Über den Sommer haben die Akteur:innen des Bündnisses das Thema eigenverantwortlich in ihren Aufgabenbereichen platziert und umgesetzt.

### Infobox Mehr Informationen zum Thema Hitzeschutzmaßnahmen in Gesundheitseinrichtungen

- Musterhitzeschutzpläne für Krankenhäuser und Arztpraxen: https://hitzeschutz-berlin.de/hitzeschutzplaene/
- Kurzschulungs- und Informationsmaterialien zum Thema Hitze: https://hitzeschutz-berlin.de/schulungsmaterial/
- KLUG-Hitzeportal für Gesundheitsberufe: https://hitze.info/

## Rolle der Pneumolog:innen

Angesichts des erhöhten gesundheitlichen Risikos von Patient:innen mit Lungen- und Atemwegserkrankungen bei hohen Temperaturen und während Hitzewellen sind auch Pneumolog:innen gefragt, in dieser Hinsicht Verantwortung zum Schutz vulnerabler Gruppen zu übernehmen. In Vorbereitung auf heißere Sommer und häufigere Hitzewellen ist es wichtig, dass Pneumolog:innen über hitzeassoziierte Erkrankungen, ihre Behandlung und Prävention informiert sind und ggf. auch Kolleg:innen dafür sensibilisieren.

Ausgewählte Maßnahmen zum Hitzeschutz der Patient:innen, aber auch der Mitarbeiter:innen in der eigenen Praxis oder Abteilung können im eigenen Handlungsbereich umgesetzt werden. So können stationär tätige Ärzt:innen etwa abteilungsspezifische Hitzeschutzpläne oder sogar klinikweite Maßnahmen anregen. Niedergelassene Pneumolog:innen können analog die Umsetzung in ihrer eigenen Praxis prüfen. Pneumolog:innen, aber auch Hausärzt:innen können ihre Patient:innen mit respiratorischen Erkrankungen beratend auf den Sommer und auf hohe Temperaturen vorbereiten. Dies schließt Verhaltensempfehlungen zur Vermeidung starker Hitzeexposition sowie eine mögliche Anpassung der Medikation ein. Besonders gefährdete Patient:innen können während Hitzeperioden gesondert kontaktiert und begleitet werden. Bei auftretenden Beschwerden an heißen Tagen und während Hitzewellen sind die spezifischen gesundheitlichen Auswirkungen von Hitze zu bedenken, insbesondere auch im Zusammenspiel mit Luftverschmutzung. Weitere Maßnahmen sind eher organisatorischer Natur und betreffen den Behandlungs- und Praxisablauf, wie beispielsweise das Einbestellen von gefährdeten Patient:innen in den kühleren Morgenstunden oder das Vermeiden von elektiven Eingriffen während Hitzewellen. Auch strukturelle Maßnahmen in Vorbereitung auf den Sommer, wie etwa die Montage von Beschattungsvorrichtungen, sind möglich.

Pneumolog:innen können einen maßgeblichen Beitrag zu gesundheitsbezogenem Hitzeschutz leisten

Da Hitze bereits heute ein Risiko für Patient:innen mit Lungen- und Atemwegserkrankungen darstellt, ist die Priorisierung von schnell umsetzbaren Maßnahmen wichtig. Baulich-technische Maßnahmen in Versorgungseinrichtungen gehören zum umfassenden Hitzeschutz dazu, benötigen aber Zeit und finanzielle Ressourcen. Insbesondere in einem Gesundheitssystem, das ohnehin bis zum Anschlag und darüber hinaus belastet und gleichzeitig mit anderen Krisen wie etwa der COVID-19-Pandemie konfrontiert ist, kann die zusätzliche Berücksichtigung von Hitze eine Herausforderung darstellen (s. dazu auch [5]). Da Gesundheitsrisiken durch Hitze in Zukunft jedoch voraussichtlich zunehmen werden, nimmt eine gute Vorbereitung spätere Arbeit ab und schützt die eigene Produktivität und Gesundheit.

## Aus‑, Fort- und Weiterbildung im Fachbereich

Um während Hitzewellen adäquat handeln zu können, ist die Verankerung diesbezüglicher Kenntnisse in der Aus‑, Fort- und Weiterbildung notwendig. Lehrkrankenhäuser sollten daher bereits Medizinstudierende sensibilisieren, in Krankenhäusern und Praxen sollte der Schulungsbedarf überprüft und ggf. sollten entsprechende Fortbildungen angeboten werden. Dabei sollten auch nichtärztliche Berufsgruppen eingebunden werden, die eine wesentliche Rolle bei Hitzeschutzmaßnahmen übernehmen können.

Für die adäquate Handlung in Hitzewellen ist die Verankerung in der Aus‑, Fort- und Weiterbildung notwendig

Die Ärzt:innenschaft sollte sich klar zum Hitzeschutz bekennen und Hitze eine höhere Priorität einräumen. Hitzeschutz gehört auf die Agenda in sämtlichen ärztlichen Verantwortungsbereichen, sei es in der Patientenversorgung, in Fachgesellschaften, Berufsverbänden oder Ärztekammern.

## Fazit für die Praxis

- Klimawandelbedingt kommt es in Deutschland zu mehr heißen Tagen sowie häufigeren, längeren und intensiveren Hitzewellen.
- Für Patient:innen mit Atemwegserkrankungen ist Hitze mit einem erhöhten Risiko für Morbidität und Mortalität verbunden.
- Es ist wichtig, dass sich Pneumolog:innen über die Risiken von Hitze und Präventionsmöglichkeiten informieren.
- Pneumolog:innen können ihre vulnerablen Patient:innen durch angepasste Beratung, Begleitung und Behandlung vor hitzebedingten Gesundheitsauswirkungen schützen.
- Die Verankerung des Themas „gesundheitsbezogener Hitzeschutz“ in der Aus‑, Fort- und Weiterbildung von Pneumolog:innen ist essenziell.
